# Identification of uncommon oral yeasts from cancer patients by MALDI-TOF mass spectrometry

**DOI:** 10.1186/s12879-017-2916-5

**Published:** 2018-01-08

**Authors:** Narges Aslani, Ghasem Janbabaei, Mahdi Abastabar, Jacques F. Meis, Mahasti Babaeian, Sadegh Khodavaisy, Teun Boekhout, Hamid Badali

**Affiliations:** 1 Student Research Committee, Mazandaran University of Medical Sciences, Sari, Iran; 2 Department of Medical Mycology and Parasitology, School of Medicine, Mazandaran University of Medical Sciences, Sari, Iran; 3 Gastrointestinal Cancer Research Center, Mazandaran University of Medical Sciences, Sari, Iran; 4 Department of Medical Microbiology and Infectious Diseases, Canisius-Wilhelmina Hospital (CWZ), Nijmegen, The Netherlands; 5 Center of Expertise in Mycology Radboudumc/CWZ, Nijmegen, The Netherlands; 6 Department of Medical Mycology and Parasitology, Tehran University of Medical Sciences, Tehran, Iran; 7 Westerdijk Fungal Biodiversity Institute, Utrecht, The Netherlands; 8 Institute of Biodiversity and Ecosystem Dynamics (IBED), University of Amsterdam, Amsterdam, The Netherlands; 9 Invasive Fungi Research Center (IFRC), School of Medicine, Mazandaran University of Medical Sciences, Sari, Iran

**Keywords:** MALDI-TOF MS, *Candida* species, Oral cavity, Cancer patients

## Abstract

**Background:**

Opportunistic infections due to *Candida* species occur frequently in cancer patients because of their inherent immunosuppression. The aim of the present study was to investigate the epidemiology of yeast species from the oral cavity of patients during treatment for oncological and haematological malignancies.

**Methods:**

MALDI-TOF was performed to identify yeasts isolated from the oral cavity of 350 cancer patients. Moreover, antifungal susceptibility testing was performed in according to CLSI guidelines (M27-A3).

**Results:**

Among 162 yeasts and yeast-like fungi isolated from the oral cavity of cancer patients, *Candida albicans* was the most common species (50.6%), followed by *Candida glabrata* (24.7%), *Pichia kudriavzevii* (*Candida krusei* (9.9%)), *Candida tropicalis* (4.3%), *Candida dubliniensis* (3.7%), *Kluyveromyces marxianus* (*Candida kefyr* (3.7%)) and *Candida parapsilosis* (1%). In addition, uncommon yeast species i.e., *Saprochaete capitata*, *Saccharomyces cerevisiae*, *Clavispora lusitaniae* (*C. lusitaniae*) and *Pichia kluyveri* (*C. eremophila*) were recovered from oral lesions. Oral colonization by *C. albicans*, non-*albicans Candida* species and uncommon yeasts were as follow; 55%, 44% and 1%, whereas oral infection due to *C. albicans* was 33.3%, non-*albicans Candida* species 60.6%, and uncommon yeasts 6.1%. Poor oral hygiene and xerostomia were identified as independent risk factors associated with oral yeast colonization. The overall resistance to fluconazole was 11.7% (19/162). Low MIC values were observed for anidulafungin for all *Candida* and uncommon yeast species.

**Conclusions:**

This current study provides insight into the prevalence and susceptibility profiles of *Candida* species, including emerging *Candida* species and uncommon yeasts, isolated from the oral cavity of Iranian cancer patients. The incidence of oral candidiasis was higher amongst patients with hematological malignancies. The majority of oral infections were caused by non-*albicans Candida* species which were often more resistant to anti-fungal agents. Our findings suggest that anidulafungin should be used as antifungal of choice for prophylaxis in clinically high-risk patients with documented oral colonization or infection.

## Background

Worldwide the incidence of mild to severe fungal infections (FI) has dramatically increased in the last several decades, especially in patients infected with human immunodeficiency virus (HIV), those that prolongedly use broad-spectrum antibiotics, patients undergoing hematopoietic stem cell transplantation (HSCT), and those receiving intensive chemo- and /or radiotherapy [1, 2]. The latter factors are important, not only because of the cytotoxic effects they have on mucosal immune defense mechanisms, but also because they change the physiology and microbial ecology of the oral environment to prolonged xerostomia and hyposalivation leading to intra-oral colonization, thus facilitating overgrowth of fungal pathogens leading to significant patient morbidity [2, 3]. The most predominant yeasts isolated from oral colonization and infection are *Candida* species that are the most important opportunistic fungal pathogens in humans and cause mucosal to deep-seated candidiasis [4]. The incidence of oral colonization and infection has been reported to be 43% to 90% and 13% to 52% among cancer patients, respectively, and involves the mouth cavity and surrounding soft tissue, followed by pain, erythema, ulceration, quantitative and qualitative salivary changes and taste disorders [5, 6]. Studies have demonstrated that *Candida albicans* (78%) is predominantly involved in oral mucositis. The species remains a major source of illness during immunosuppression despite the application of antifungal therapy and can cause a systemic infection associated with significant morbidity and mortality rates [3, 7]. Although *C. albicans* is responsible for the vast majority of oral colonizations and infections*,* non-*albicans Candida* species belong to diverse species complexes, i.e., *Candida parapsilosis*, *Candida glabrata*, *Candida tropicalis*, *Kluyveromyces marxianus* (*Candida kefyr*), *Pichia kudriavzevii* (*Candida krusei*), *Meyerozyma guilliermondii* (*Candida guilliermondii*) and other uncommon yeast species with a reduced susceptibility to triazoles and echinocandins become a serious clinical challenge and thus the isolates need to be properly identified [8–12]. Little is known about the etiological importance of yeasts in oral colonization and infection among Iranian cancer patients. Identification using DNA barcodes, is time consuming, expensive and sometimes unspecific for cryptic species. Matrix-assisted laser desorption/ionization time-of-flight mass spectrometry (MALDI-TOF MS) is an important technique for the rapid and reliable identification of species with sufficient specificity, reproducibility and sensitivity [13, 14]. Herein, we investigated the prevalence of *Candida* species and uncommon yeasts responsible for colonizing and infecting the oral cavity of patients with different types of cancer using MALDI-TOF MS for identification. Moreover, guidelines for the treatment of candidiasis have approved the use of polyenes, azoles and echinocandins [15, 16]. Notably, in recent years, highly elevated MICs to antifungals, especially fluconazole, have appeared and antifungal drug resistance is quickly becoming a serious concern, especially when immunological defenses are impeded. In vitro antifungal susceptibility tests have an important role in surveillance of resistance and are also beneficial for choosing the right antifungal agents to be used for treatment. It seems that non-*albicans Candida* strains show considerable emergence of resistance to antifungal drugs [10, 17, 18]. Limited data on the in vitro antifungal susceptibility patterns against isolates of *Candida* species from the oral cavity of patients with oncological and hematological malignancies are available. Therefore, in vitro antifungal susceptibilities were determined against 162 clinically isolates of *Candida* species and uncommon other yeast species from Iranian cancer patients.

## Methods

This study was performed at the cancer center of the Mazandaran University Hospital that has 1600 oncology and hematology in-patient admissions per year. All patients were required to sign an informed consent form prior to entry into the study. During one year 350 oral swab samples were randomly obtained from Iranian cancer patients undergoing intensive chemotherapy, namely 210 (60%) males and 140 (40%) females and ranging 17–88 years in age with and without sign and symptoms of oral infection, including white plaque, erythematous and ulcerative lesions, dryness, pain, altered taste sensation and halitosis. Demographic data on age, gender, oral hygiene, type of cancer, history of prior fungal infections, use of topical or systemic antifungal therapy, prophylaxis and medications were collected at enrolment.

### Study design and clinical specimens

As oral examination, signs and symptoms of inflammation suggestive of oral candidiasis were documented and included soreness, erythema, ulceration and the presence or absence of white plaques in the mouth. Specimens were obtained by sterile cotton swabs moistened with normal saline that were placed on the tongue, buccal mucosa and labial sulcus, transported in sterile tubes and were examined initially in 10% KOH, followed by inoculation on malt extract agar (MEA, Difco) supplemented with chloramphenicol and CHROMagar *Candida* medium (CHROMagar Company, Paris, France) to ensure purity, and incubated at 37 °C for 24 h. Strains were preliminarily identified using conventional methods, i.e., germ tube tests, formation of pseudohyphae, production of chlamydospores and carbohydrate assimilation tests using the ID 32 C kit (bioMérieux, Marcy l’Etiole, France). Oral yeast colonization was defined as presence of yeasts in the oral cavity. Oral candidiasis was defined as presence of *Candida* species in the oral cavity together with symptoms and signs of inflammation ⁄ mucositis and ⁄ or presence of white plaques.

### Fungal species identification

Subsequently, genomic DNA was extracted from all test isolates along with reference strains as described by Xu et al. and stored at −80 °C prior to use [19]. All strains were identified by PCR-RFLP and using the amplified HWP1 gene to distinguish members of the *C. albicans* complex as previously described [20]. Cultures of *Candida* isolates were preserved at −70 °C at the reference culture collection of invasive fungi research center (IFRC, Sari, Iran) using cryo-tubes (Mast Diagnostics, Bootle, Merseyside, UK) until further analysis.

MALDI-TOF MS-based identification of all yeast and yeast-like isolates was performed according to Bruker Daltonics (Bremen, Germany) with the ethanol (EtOH)/formic acid (FA) extraction protocol. For extraction, two loops of yeast biomass (1-μvolume, sterile inoculation loop) not older than 24 h growing on Sabouraud Dextrose Agar (SDA) were suspended in 300 μl of molecular graded ionized water and further processed with 900 μl of 95% EtOH. A volume of 25 μl of FA was found to be optimal and an equal volume of acetonitrile (ACN) was added later. From the crude protein extract of each tested strain, 1 μl was spotted onto steel target plate (Bruker Daltonics) and allowed to dry at room temperature. Bacterial test standard (Bruker Daltonics) was used as a positive control. Before measurements, all tested spots were overlaid with 1 μl of alpha-cyano-4- hydroxycinnamic acid (Bruker Daltonics) saturated matrix solution. The yeast identification was operated by the MALDI Biotyper 3.0 system based on mass spectra generated with the Microflex LT software and compared with two databases simultaneously. Identification scores were interpreted according to the manufacturer’s recommended criteria: a log score value >2.0 indicated correct identification to the species level, a log score 1.999 > value >1.7 correct genus recognition, and no reliable identification with a score < 1.7. Each isolate was considered correctly identified if at least one of the duplicates gained scores >2. Strains with results <2.0 or no peaks found were retested from a fresh culture. The identification was also considered correct if at least one spot from the duplicate gave a reliable identification with score > 1.7 that was concordant with the sequencing results [21, 22].

### Antifungal susceptibility testing

Antifungal susceptibility testing was carried out using the Clinical and Laboratory Standards Institute broth microdilution method (CLSI), following the M27-A3 and M27-S4 guidelines [23, 24]. Amphotericin B (AMB; Sigma, St. Louis, MO, USA), fluconazole (FLU; Pfizer, Groton, CT, USA), caspofungin (CFG; Merck Sharp and Dohme BV, Haarlem, The Netherlands) and anidulafungin (AFG; Pfizer) were dissolved in 1% dimethyl sulfoxide (DMSO, Sigma) and were prepared at a final concentration of 0.016–16 μg/ml for amphotericin B; 0.063–64 μg/ml for fluconazole, and 0.008–8 μg/ml for caspofungin and anidulafungin. RPMI 1640 medium with glutamine without bicarbonate (Sigma) buffered to pH 7 with 0.165 mol/l 3-N-morpholinepropanesulfonic acid (MOPS; Sigma) was used. Drug-free and yeast-free controls were included, and microtiter plates were incubated at 35 °C and read visually after 24 h, as validated recently by Pfaller et al. [25, 26]. *Pichia kudriavzevii* (= *C. krusei*) ATCC 6258 and *Candida parapsilosis* ATCC 22019 were used as quality control strains. Except for amphotericin B, the MIC endpoints for all antifungals were defined as the lowest drug concentration that caused 50% growth inhibition *vis-à-vis* the drug-free controls. The MIC for amphotericin B was defined as the lowest concentration at which there was 100% inhibition of growth.

### Statistical analysis

Statistical analyses were performed using SPSS (version 24.0; Windows, Chicago, IL, USA) and R Software (version 3.0.1). Binary and bayesian logistic regression were used to model the effect of risk factors on colonization. *P* values of <0.05 were considered statistically significant.

## Results

**Fig. 1:**
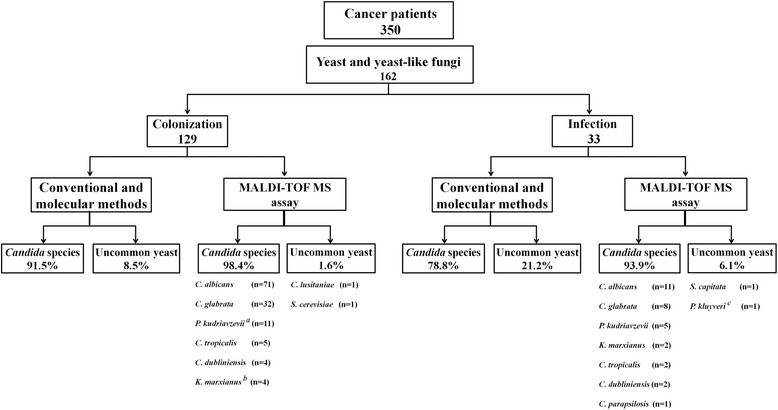
Flow diagram for the identification of 162 yeast and yeast-like isolates. ^a^ *Pichia kudriavzevii* (*Candida krusei*). ^b^ *Kluyveromyces marxianus* (*Candida kefyr).* ^c^ *Pichia kluyveri* (*Candida eremophila*)

**Fig. 2:**
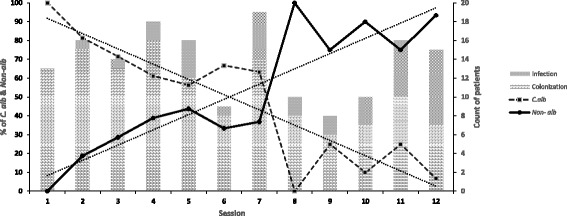
Distribution of oral colonization and infection by *C. albicans* and non*-albicans Candida* among cancer individuals

Demographic, clinical information and types of cancers in the study population are summarised in Table 1. In total, we collected 162 (46.3%) strains of yeasts and yeast-like fungi from 350 cancer patients undergoing intensive chemotherapy. The mean age of the patients was 53 years (range 17–88 years). Among the patients, males comprised 58.6% (95/162) and females 41.4% (67/162). Nearly half (*n* = 83, 51.2%) of the patients suffered from different types of lymphoma, 18 (11%) had colorectal cancer, 13 (8%) lung cancer, 13 (8%) gastric cancer, 10 (6.2%) breast cancer, and 25 (15.4%) other solid tumors (i.e. liver, kidney, nasopharyngeal, prostate, esophagus, cervical, uterus and ovaries cancer). The age of the majority of cancer patients ranged from 41 to 60 (51/162). A logistic regression analysis identified xerostomia and poor oral hygiene as independent risk factors associated with oral yeast colonization (Table 2). Oral yeast colonization was prevalent in 79.6% (129/162) of all cancer patients and 20.4% (33/162) of those had clinical and microbiological evidence of oral candidiasis. Type of cancer and the number of chemotherapy sessions showed a statistical significant risk for the development oral infection. Figure 2 shows that increasing the number of chemotherapy sessions of cancer patients, correlated with the presence oral candidiasis. Oral yeast colonization varied amongst different cancer types and was high in solid tumor patients (colonization rate 40.7%) compared with patients with hematological malignancies (38.9%). Patients with hematological malignancies had a higher rate of oral infection (12.3%) than those with solid tumors (8%) (Table 1). Figure 1 shows the results of identification based on conventional and molecular tests and their concordance at genus and species levels with MALDI-TOF assessments. Conventional and molecular methods identified 144 isolates as *C. albicans* (*n* = 82), *C. dubliniensis* (*n* = 6), *C. glabrata* (*n* = 40) and *P. kudriavzevii* (= *C. krusei*, *n* = 16). The species distribution belonging to the oral colonization was *C. albicans* (*n* = 71), *C. dubliniensis* (n = 4), *C. glabrata* (*n* = 32), *P. kudriavzevii* (*C. krusei*) (*n* = 11) and unknown species (n = 11). Overall *C. albicans* (n = 11), *C. dubliniensis* (*n* = 2), *C. glabrata* (n = 8), *P. kudriavzevii* (*C. krusei*) (*n* = 5) and unknown species (n = 7) causes of oral candidiasis were identified by conventional and molecular tests (Fig. 1). Among the *C. albicans* complex, neither *C. stellatoidea* nor *C. africana* were identified by HWP1 gene amplification. Subsequently, all isolates and the remaining 18 unknown isolates were identified and confirmed with MALDI-TOF at the species level. Our data showed that MALDI-TOF MS correctly identified 100% isolates with score > 2.0, indicating a secure genus and species identification. Isolates with scores of >2.0 comprised four genera and 11 species. Among the 162 isolates, MALDI-TOF MS identified 7 isolates (4.3% of all yeast isolates) as *C. tropicalis*, 6 (3.6% of all yeast isolates) as *K. marxianus* (= *C. kefyr*) and 1 isolate as *C. parapsilosis*, which were not detected by the conventional and PCR-RFLP based identification methods. Overall *C. glabrata* (*n* = 40, 24.7%)*,* followed by *P. kudriavzevii* (*C. krusei*) *(n* = 16, 9.9%) were the most frequent leading agent of non-*albicans Candida* species among cancer patients. Overall 47.5% of patients (*n* = 77) were colonized or infected by non*-albicans Candida* species. In addition, MALDI-TOF MS identified (n = 4, 2.5% of cancer patients) isolates as rarely uncommon yeasts, i.e., *Saprochaete capitata*, *Saccharomyces cerevisiae*, *Clavispora lusitaniae* (= *C. lusitaniae*) and *Pichia kluyveri* (= *C. eremophila*)*,* which were not detected by the PCR-RFLP method. Uncommon yeast species that colonized the oral cavity of cancer patients were *C. lusitaniae* and *S. cerevisiae,* while *S. capitata* and *P. kluyveri* were found in oral infection (Fig. 1). The majority of cancer patients were colonized or infected by a single yeast species, but 4.9% (*n* = 8) of patients had two different species present. Only patients who developed infection were given antifungal medications. Of these, 22 (13.6%) received prophylaxis with fluconazole (200 mg/kg/d for 7–14 days) and if clinical cure was not achieved in 14 days, the dose was increased up to 400 mg/d. All these 22 patients had persistent candidiasis and were culture positive for *Candida* species (*C. albicans n* = 7, *P. kudriavzevii* (*C. krusei*) *n* = 5, *C. dubliniensis n* = 3, *C. glabrata* n = 3, and one isolate of *K. marxianus* (*C. kefyr*)*, C. tropicalis*, *S. capitata* and *P. kluyveri*)*.*

**Table 1:** Demographic and clinical description of Iranian cancer patients with oral colonization or infection

Cancer Type	Count		Candidiasis / Colonization	Species		Prophylaxis
				Anamorph	Teleomorph	
	Total (%)	Sex (M/F)				
Basal Cell Carcinoma	1 (0.6%)	0/1	0/1	*C. albicans* (1)	Unknown	1
Breast Cancer	10 (6.1%)	0/10	3/7	*C. albicans* (9)	-	1
				*C. krusei* (1)	*Pichia kudriavzevii* (1965)	
Bronchial Adenoma	1 (0.6%)	1/0	0/1	*C. albicans* (1)	–	–
Congestive Heart Failure	1 (0.6%)	0/1	0/1	*C. albicans* (1)	–	–
Colorectal Cancer	18 (11.1%)	9/9	3/15	*C. albicans* (7)	-	1
				*C. glabrata* (5)	Unknown	
				*C. kefyr* (3)	*Kluyveromyces marxianus* (1971)	
				*C. dubliniensis* (2)	Unknown	
				*C. lusitaniae* (1)	*Clavispora lusitaniae*	
Nasopharyngeal Cancer	1 (0.6%)	1/0	1/0	*C. albicans* (1)	–	–
Esophagus Cancer	6 (3.7%)	2/4	0/6	*C. glabrata* (3)	-	–
				*C. albicans* (2)	-	
				*C. krusei* (1)	*Pichia kudriavzevii* (1965)	
Gastric Cancer	13 (8.0%)	8/5	2/11	*C. albicans* (8)	-	2
				*C. glabrata* (2)	-	
				*C. dubliniensis* (1)	-	
				*C. tropicalis* (1)	Unknown	
				*Saprochata capitata* (1)	Unknown	
Hepatocellular Carcinoma	5 (3.1%)	2/3	1/4	*C. albicans* (3)	-	1
				*C. glabrata* (2)	-	
Renal Cell Carcinoma	1 (0.6%)	1/0	0/1	*C. albicans* (1)	–	–
Lung Carcinoma	13 (8.0%)	10/3	3/10	*C. albicans* (10)	-	–
				*C. glabrata* (3)	-	
Lymphoma	83 (51.2%)	57/26	20/63	*C. albicans* (32)	-	16
				*C. glabrata* (22)	-	
				*C. krusei* (14)	*Pichia kudriavzevii* (1965)	
				*C. tropicalis* (6)	*-*	
				*C. dubliniensis* (3)	*-*	
				*C. kefyr* (3)	*Kluyveromyces marxianus* (1971)	
				*C. eremophila* (1)	*Pichia kluyveri*	
				*C. parapsilosis* (1)	Unknown	
				*C. robusta* (1)	*Saccharomyces cerevisiae*	
Nasopharyngeal Cancer	2 (1.2%)	2/0	0/2	*C. glabrata* (2)	–	–
Pancreatic Cancer	1 (0.6%)	1/0	0/1	*C. albicans* (1)	–	–
Prostate Cancer	1 (0.6%)	1/0	0/1	*C. albicans* (1)	–	–
Sarcoma	1 (0.6%)	1/0	0/1	*C. glabrata* (1)	–	–
Uterine Cancer	4 (2.5%)	0/4	0/4	*C. albicans* (4)	–	–
Total	162 (≈100%)	95/67	33/129	*C. albicans* (82)	-	22
				*C. glabrata* (40)	-	
				*C. krusei* (16)	*Pichia kudriavzevii* (1965)	
				*C. tropicalis* (7)	-	
				*C. dubliniensis* (6)	-	
				*C. kefyr* (6)	*Kluyveromyces marxianus* (1971)

**Table 2:** Logistic regression analysis of risk factors for oral yeast colonization

Risk factors	*P*-value	Odds ratio	95% CI
Xerostomia	0.019	4.994	1.288–18.452
Poor oral hygiene	0.014	2.734	1.207–6.261

### Antifungal susceptibility profiles

The in vitro susceptibility data and the MIC distribution are presented in Tables 3 and 4. A total of 162 isolates of *Candida* species and uncommon yeasts were analyzed for their susceptibility to fluconazole, amphotericin B, caspofungin and anidulafungin. Fluconazole exhibited no activity against 15.9% (*n* = 13) of *C. albicans* isolates (MIC of 16 to >64 μg/ml), whereas the remaining 69 isolates revealed a MIC range of 0.063–8 μg/ml. Notably, 12.5% of *C. glabrata* isolates (*n* = 5) showed fluconazole MICs of >64 μg/ml. All tested *C. albicans* isolates had low MICs of amphotericin B (MIC_50_ values of 1 μg/ml) but 35.4% (*n* = 29) of *C. albicans* isolates had markedly elevated amphotericin B MICs ranging from 2 to 16 μg/ml. Moreover, elevated geometric means (GM) were observed for caspofungin (0.34 μg/ml) in comparison to anidulafungin (0.06 μg/ml). Notably, 35% (*n* = 14) of the *C. glabrata,* 15.9% (*n* = 13) of the *C. albicans* and 75% (*n* = 12) of the *P. kudriavzevii* (*C. krusei*) isolates revealed MICs of ≥1 μg/ml to caspofungin. Overall 27.8% of *Candida* species and uncommon yeasts revealed MICs of ≥1 μg/ml to caspofungin. All isolates showed reduced MICs to anidulafungin with MICs ranging from 0.008 to 0.5 μg/ml, except four isolates of *C. glabrata* that had a MIC of 1 μg/ml (Tables 3 and 4). Overall in terms of GM MICs, anidulafungin demonstrated potent activity against all isolates (*n* = 162), in comparison with amphotericin B, caspofungin and fluconazole.

**Table 3:** In vitro susceptibilities of 157 clinical isolates to four antifungal agents. MIC range, geometric mean, MIC_50_, and MIC_90_ values are expressed in μg/ml

(No. of strains)	Antifungal agent	MIC μg/ml				
		MIC range	MIC_50_	MIC_90_	GM	MODE
All clinical strains (*n* = 157)	Fluconazole	0.063–64	2	8	1.65	4
	Amphotericin B	0.016–16	1	4	0.50	1
	Caspofungin	0.008–8	0.5 mg/ml	2 μg/ml	0.34	0.5
	Anidulafungin	0.008–1	0.063	0.5	0.06	0.063
*C. albicans* (*n* = 82)	Fluconazole	0.063–64	0.5	8	0.80	0.5
	Amphotericin B	0.016–16	1	4	0.62	1
	Caspofungin	0.008–8	0.25	1	0.22	0.5
	Anidulafungin	0.008–0.25	0.031	0.125	0.03	0.016
*C. dubliniensis* (*n* = 6)	Fluconazole	0.063–0.125	0.125	0.125	0.11	0.125
	Amphotericin B	0.016–2	0.031	2	0.09	0.031
	Caspofungin	0.25–2	0.5	2	0.5	0.5
	Anidulafungin	0.008–0.125	0.125	0.25	0.07	0.016
*C. glabrata* (*n* = 40)	Fluconazole	0.25–64	8	64	7.08	4
	Amphotericin B	0.016–4	1	2	0.62	1
	Caspofungin	0.008–2	0.5	2	0.40	1
	Anidulafungin	0.016–1	0.063	1	0.12	1
*C. krusei (= Pichia kudriavzevii)* (*n* = 16)	Fluconazole	0.25–64	8	64	7.33	4
	Amphotericin B	0.063–2	0.5	1	0.59	1
	Caspofungin	0.063–4	2	4	1.13	1
	Anidulafungin	0.016–0.25	0.125	0.25	0.07	0.25
*C. tropicalis* (*n* = 7)	Fluconazole	0.063–8	4	8	0.63	0.063
	Amphotericin B	0.031–2	1	2	0.39	2
	Caspofungin	0.031–8	0.5	8	0.39	0.25
	Anidulafungin	0.008–0.063	0.063	0.063	0.063	0.063
*C. kefyr = (Kluyveromyces marxianus)* (*n* = 6)	Fluconazole	0.25–32	4	32	2.82	4
	Amphotericin B	0.016–1	0.5	1	0.15	0.5
	Caspofungin	0.125–0.05	0.25	0.5	0.25	0.125
	Anidulafungin	0.031–0.063	0.063	0.5	0.04	0.063

**Table 4:** In vitro susceptibilities of rare clinical species (n = 1) to four antifungal agents

Strains	Antifungal agent	MIC (μg/ml)
*C. parapsilosis*	Fluconazole	0.25
	Amphotericin B	0.125 μg/ml
	Caspofungin	4
	Anidulafungin	0.25
*Pichia kluyveri* (*Candida eremophila*)	Fluconazole	0.5
	Amphotericin B	1
	Caspofungin	4
	Anidulafungin	0.25
*Clavispora lusitaniae* (*Candida lusitaniae*)	Fluconazole	0.5
	Amphotericin B	0.016
	Caspofungin	2
	Anidulafungin	0.25
*Saccharomyces cerevisiae*	Fluconazole	0.5
	Amphotericin B	0.016
	Caspofungin	4
	Anidulafungin	0.25
*Saprochaete capitata*	Fluconazole	1
	Amphotericin B	4
	Caspofungin	0.125
	Anidulafungin	0.016

## Discussion

Fungal infections caused by *Candida* species are common in immunocompromised patients, and the incidence has dramatically increased during the last decades [27, 28]. Several virulence factors contribute to the pathogenicity of *Candida* yeasts, which is one of the main causes of systemic infection in individuals with cancer and contribute to high mortality rates of these patients [29]. Previous studies reported an incidence of oral yeast colonization and infection amongst cancer patients, ranging from 43% to 90% and 13% to 52%, respectively, depending greatly on the type of cancer, regimens to manage the *Candida* infections, and advance of the disease [6]. In our study we have investigated and compared the yeast colonization and infection rate amongst Iranian cancer patient groups, namely those with hematological malignancies and solid tumors during chemotherapy, as well as their antifungal susceptibilities. On sub-analysis of our study data, it becomes apparent that there is a distinct difference in colonization and infection rate and occurrence of yeast species amongst different types of cancer and different chemotherapy sessions (Table 1 and Fig. 2). The oral yeast colonization rate was 79.6% and the incidence of oral infections (20.4%) in our cancer population, which are similar to other studies [6, 30]. Rautema et al. and Schelenz et al. observed similar rates of oral candidiasis during radiotherapy treatment, but this is much lower compared with previously reported studies that reported rates of up to 58% [6, 31, 32]. It is well known that chemo-radiotherapy can lead to mucositis, xerostomia and damage of the cell mediated immunity which plays an important role in the pathogenesis of oral candidiasis and lead to promotes manifestation of yeast infection [33, 34]. The oral colonization rate of our hematology patients (38.9%) had little difference compared with other studies in cancer patients (49.4% and 41%) [6, 32]. A low oral infection rate (8%) was seen in patients with solid tumors although this group had somewhat higher yeast colonization (40.7%). Patients with hematological malignancies who generally undergo more aggressive chemotherapy leading to a higher degree of generalized immunosuppression, disruption of the mucosal barrier have a subsequent increased risk of infection when compared with solid tumor patients, such as those with prostate cancers. In this group of patients, it is important to emphasize good oral hygiene and prevention of infection. Our study showed that xerostomia and poor oral hygiene are independent risk factors associated with an increased risk of oral yeast colonization. Such association has previously been described in hospitalized elderly patients and is often accompanied with age, compromised nutritional status, presence of dental prosthesis, hyposalivation, and terminal illness [4, 6, 35]. Several studies have analyzed which *Candida* species were involved in colonization and infection of cancer patients. A study in Brazil showed that nearly half of the patients (42.9%) were colonized by *C. albicans* and 33% of the patients by non-*albicans* species (*C. tropicalis*, *P. kudriavzevii* (*C. krusei*) and *C. lusitanae*) [36]. The prevalence of *Candida* species in our study is similar to those mentioned in other reports [37–39]. A recent study by Schelenz et al. reported the distribution of *Candida* species in 400 patients suffering from hematological malignancies, head neck cancers and solid tumors. These authors found *C. albicans* (74%) species followed by *C. glabrata* (11.5%), *C. tropicalis* (2.6%), *C. krusei* (2.6%) and *C. parapsilosis* (1.9%) to be involved [6]. Another study showed that approximately 85% (*n* = 50) of cancer patients were positive for culture of *Candida* species from the oral mucosa, with *C. albicans* being the most prevalent species, followed by *C. glabrata* with 14.5% of cancer patients [29]. Unlike other reports, the uncommon species of *S. capitata* and *P. kluyveri* were found to be involved in oral colonization and infection in the current study (Table 1). Other species, including *S. cerevisiae*, *Debaryomyces hansenii* (= *C. famata*) and *Clavispora lusitaniae* (*= C. lusitaniae*), have been previously isolated from oral specimens, thereby acting as potential pathogens of oral infection [6, 37]. Similar to other studies, the co-isolation were seen in 4.9% (*n* = 8) of our cancer patients. [6, 37]. Laboratories should be able to detect mixtures of different yeast species in primary cultures because it is an important issue for the management of oral candidiasis patients. In this study emerging species recovered from oral cavity of cancer patients such as *K. marxianus* (*C. kefyr*), *C. parapsilosis*, *C. tropicalis* and *P. kudriavzevii* (*C. krusei*) were misidentified by conventional methods. This finding may be related to the fact that more reliable techniques are rarely used to identify these species from clinical samples so far. Simple PCR-based analysis using amplicon length differences has been used to identify closely related *Candida* species in several studies [40]. Recent studies described the use of MALDI-TOF mass spectrometry to discriminate clinical samples [17, 41]. Accordingly, in this study, the simultaneous use of PCR assays and MALDI-TOF MS turned out to be a powerful technique for the accurate identification of yeasts isolated from the oral cavity. These findings are in accordance with recent studies showing yeast identification rates of 97.6% by MALDI-TOF MS [21, 42]. Similar to other studies, it seems that the incidence of oral candidiasis in cancer patients showed a shift towards non*-albicans* species probably due to chemotherapy complications, the use of azoles as empirical treatment, or azole prophylaxis [43]. Remarkably, the emergence of non-*albicans* species was associated with reduced susceptibility to azole antifungal agents it compared with these of common *Candida* species [18]. A serious concern in cancer patients is that drug resistant isolates may invade the deeper tissues leading to disseminated infection. Therefore, according to the availability of limited data on in vitro antifungal susceptibility values, determination of the antifungal resistance pattern is highly essential before deciding on a specific treatment in order to choose the proper antifungal drug and to predict the outcome of therapy for routine periodic surveillance of fungal infections [11]. Fluconazole is the drug of choice for prophylaxis and treatment of severe candidiasis in cancer patients, but prolonged use of this agent has contributed to the development of drug resistance in *Candida* isolates [44, 45]. The resistance rate for fluconazole in the present study was 15.9% and 12.5% for *C. albicans* and *C. glabrata,* respectively, which is in concordance with other studies [37, 39, 46, 47]. A sub-analysis of our 162 isolates showed that resistance to fluconazole was higher (11.7%) compared with an UK-based study that indicated a lower resistance rate for fluconazole (4.5%) [6]. All of the uncommon yeasts were susceptible to fluconazole. In our study, 39% of yeast strains from cancer patients exhibited higher MIC values to amphotericin B, with MIC_90_ values of 4 μg/ml. Regarding echinocandins, 27.8% of the *Candida* isolates were resistant to caspofungin, while all isolates, except *C. glabrata* (10%), were highly susceptible to anidulafungin. Unfortunately, we did not have information of recruited cancer patient prior to the initiation of chemotherapy and/or radiotherapy. This could be seen as a comparative data of the effect of chemotherapy and/or radiotherapy on epidemiology of *Candida* spp.

## Conclusions

In conclusion, the results showed that non-*albicans* species and uncommon yeasts are the most common yeast isolates obtained from oral infections from Iranian cancer patients, and *C. glabrata* and *P. kudriavzevii* (*C. krusei*) generally predominate. Overall, the reasons for the emergence of the rare yeast species are not clear, but some medical conditions such as an increase of the number of chemotherapy sessions, the use of azoles as empirical treatment or azole prophylaxis may increase the risk of developing infection caused by these uncommon yeasts. Furthermore, these increases may also be attributed to improvement in the diagnostic methodology e.g., use of MALDI-TOF MS, making it possible to identify such uncommon yeast species. Second, a significant proportion of isolates demonstrated reduced susceptibility to currently used antifungal agents. The study indicates that anidulafungin would be the drug of choice in cancer patients undergoing intensive chemotherapy with documented oral colonization or candidiasis. However, the clinical effectiveness in the treatment of infection and colonization remains to be determined.

## Abbreviations

AFG: Anidulafungin
AMB: Amphotericin B
CAN: Acetonitrile
CFG: Caspofungin
CHROMagar: CHROMagar Candida medium
CLSI: Clinical and Laboratory Standards Institute broth microdilution method
DMSO: Dimethyl sulfoxide
EtOH: Ethanol
FA: Formic acid
FI: fungal infections
FLU: Fluconazole
GM: Geometric means
HIV: Human Immunodeficiency virus
HSCT: Hematopoietic stem cell transplantation
Hwp1: Hyphal wall protein 1
IFRC: Invasive fungi research center
MALDI: Matrix-assisted laser desorption/ionization
MEA: Malt extract agar
MIC: The minimum inhibitory concentration
MOPS: 3-N-morpholinepropanesulfonic acid
MS: Mass spectrometry
PCR-RFLP: Polymerase chain reaction - restriction fragment length polymorphism
RPMI: Roswell Park Memorial Institute medium
SDA: Sabouraud Dextrose Agar
TOF: Time-of-flight mass
